# Evaluation of Hygrothermal Behaviour in Heritage Buildings through Sensors, CFD Modelling and IRT

**DOI:** 10.3390/s21020566

**Published:** 2021-01-14

**Authors:** Carlos Lerma, Júlia G. Borràs, Ángeles Mas, M. Eugenia Torner, Jose Vercher, Enrique Gil

**Affiliations:** 1 Department of Architectural Constructions, Universitat Politècnica de València, Camino de Vera s/n, 46022 Valencia, Spain; jugarbor@csa.upv.es (J.G.B.); amas@csa.upv.es (Á.M.); jvercher@csa.upv.es (J.V.); 2 Department of Continuous Medium Mechanics and Theory of Structures, Universitat Politècnica de València, Camino de Vera s/n, 46022 Valencia, Spain; meutorfe@upvnet.upv.es (M.E.T.); egil@upv.es (E.G.)

**Keywords:** CFD, IRT, natural convection, building materials, heritage

## Abstract

Architectural heritage, building materials and interior space are highly susceptible to temperature and relative humidity. A better knowledge of the hygrothermal dynamics inside buildings allows an adequate conservation of heritage. This work compares three non-destructive techniques (NDT), such as temperature and relative humidity sensors, finite element simulations (CFD) and thermographic pictures (IRT). The work has made it possible to carry out an assessment of the risk of condensation over a year and to identify affected periods and areas of the building. Sensors and IRT pictures provide real data to validate CFD simulations, facilitating a global analysis of the building. The results provided reflect a great concordance between the NDTs used.

## 1. Introduction

Heritage conservation focuses its efforts on the early detection of pathology. Knowing climatic parameters inside buildings and the response of construction materials helps to have a better understanding of reality. The development of non-destructive tests (NDT) are widely used [1,2,3,4,5,6], since they provide information on real cases and allow analysis and diagnosis [7], as well as a monitoring tool of the work’s progress and the assessment of materials and rehabilitation interventions [8]. The goal is preventive conservation while minimizing damage [9,10]. In this sense, various types of sensors or instruments have been used to monitor this type of building [11,12,13,14,15,16].

In buildings with natural ventilation, the air movement only depends on wind speed and indoor–outdoor temperature gradient [17]. High relative humidity and low temperatures are conditions that commonly occur in heritage buildings such as churches [18]. The study of the conditions inside the churches has been analysed to know the behaviour as a function of the external conditions and the construction materials [19]. A multidisciplinary approach on historic building microclimate facilitates the identification of the main causes of deterioration in the heritage and allows to suggest conservation strategies [20]. Also, monitoring and analysing microclimate with the environmental conditions would be suitable for preservation of historical buildings as a result of thermodynamic simulation [21]. On the one hand, variations in temperature and relative humidity damage construction materials [22,23]. On the other hand, the ventilation rate due to user behaviour, window-opening habits and others must also be considered [24].

It is a great novelty to use a finite element software for modelling using Computational Fluid Dynamics (CFD) by applying it to the inside of a building to study the hygrothermal behaviour. Thermal calculations have already been made with finite elements in specific parts of buildings, such as in the encounter between enclosures, slabs and cantilevers [25]. Simulations were performed with the software Ansys Fluent 2020 R2. The parameters and characteristics of the simulations were carried out in accordance with the software application manual [26]. This program has been widely used in other branches of engineering, for the simulation of vehicle behaviour (Formula 1 cars, airplanes...), but also for buildings and heritage [27,28], as well as ventilation in buildings [29,30,31].

However, CFD and sensors, together with Infrared Thermography (IRT), have not been used in combination so far. Non-destructive techniques, such as IRT, facilitate the study of materials and allow understanding and deepening the understanding of materials and construction processes, without the need to cause damage to the building. IRT has been widely used for the protection of heritage [32] and information about the materials is provided, as well as the factors, processes and state of deterioration, and the detection of defects [33,34]. A combination of simplified and advanced analytical methods allows to perform predictive analysis of behaviour [35,36], along with the monitoring of building materials and identification of their pathology [37,38].

This study combines three non-destructive techniques (sensors, CFD and IRT) and compares the results obtained. The proposed methodology seeks to determine those areas of the buildings that are susceptible to deterioration of the materials. The risk of condensation, which depends on certain conditions of relative temperature and humidity, is evaluated. The real data from the sensors and from the thermographic pictures allow to validate the simulations carried out with the CFD model of the building.

## 2. Materials and Methods

The objective pursued in this research is to demonstrate the correlation between real data and computer simulations in order to validate the latter. The scope of study has focused on the analysis of temperature and humidity inside a heritage building. The real data of temperature and relative humidity have been provided by sensors distributed throughout the building. Infrared thermography has been used to complete this analysis with real temperature data.

The real data can be compared with those obtained by simulations and, where appropriate, validated. The simulations cover the entire volume of the building, so their subsequent study allows to obtain a better understanding of the hygrothermal dynamics. Thus, it is possible to identify critical situations or areas susceptible to condensation and, therefore, deterioration of construction materials.

### 2.1. Case Study

To carry out this research, a four-century-old heritage building was chosen, one of the most important Baroque temples among the architectural examples of this period in Spain [39,40,41,42]. It is a representative example of the medieval churches of the 17th century in Valencia (Spain) and a good part of the European territory [43,44]. It is the Church of *La Asunción de Llíria*, built between 1626 and 1783 [45]. These buildings have thick façade walls, a large volume of air inside and few ventilation and lighting holes. In addition, they have a very specific (religious) use concentrating a significant number of people at very specific times (masses).

**Figure 1:**
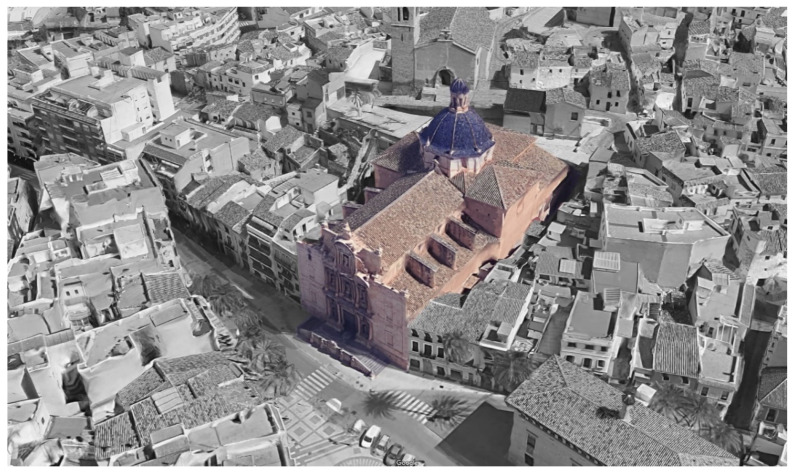
Aerial view of the building (Source: Google & National Geographic Institute of Spain).

The location of the building is unique since it is partially excavated in the mountain and has annex buildings (Figure 1). The building consists of a central nave, two sides with chapels between buttresses, an altar, a transept with a dome, as well as other rooms (offices, warehouses).

### 2.2. Real Data with Sensors

Temperature and relative humidity data have been recorded from August 2019 to August 2020. Data has been stored for 13 months so that the building’s response can be observed in all seasons. Likewise, it has coincided with periods of confinement and mobility restrictions for people derived from the Sars-Cov-2 virus and the COVID-19 disease, leaving the building without any activity and without human presence for several weeks between March and June 2020.

For the acquisition of temperature and humidity data, six low-cost data loggers from Perfec-Prime [46] were used, whose main characteristics are shown in Table 1. The recommendations of the European standard EN 15758 about the conservation of cultural property, procedures and instruments for measuring temperatures of the air and surfaces of objects have been followed [47].

The sensors are autonomous (Battery: 3 V, AAA) and the values have been recorded every 30 min. Current sensors allow the recording of values between 1 day and 1 min, but more frequently a large amount of information is generated, and it is necessary to store it [48]. Every 30 min it is possible to obtain the temperature wave along the day with great precision and with an excellent battery performance.

A total of 109,478 records have been reached. Each record includes the value of temperature, relative humidity, date, time, and sensor number. This assumes more than 200,000 values of temperature and relative humidity.

**Figure 2:**
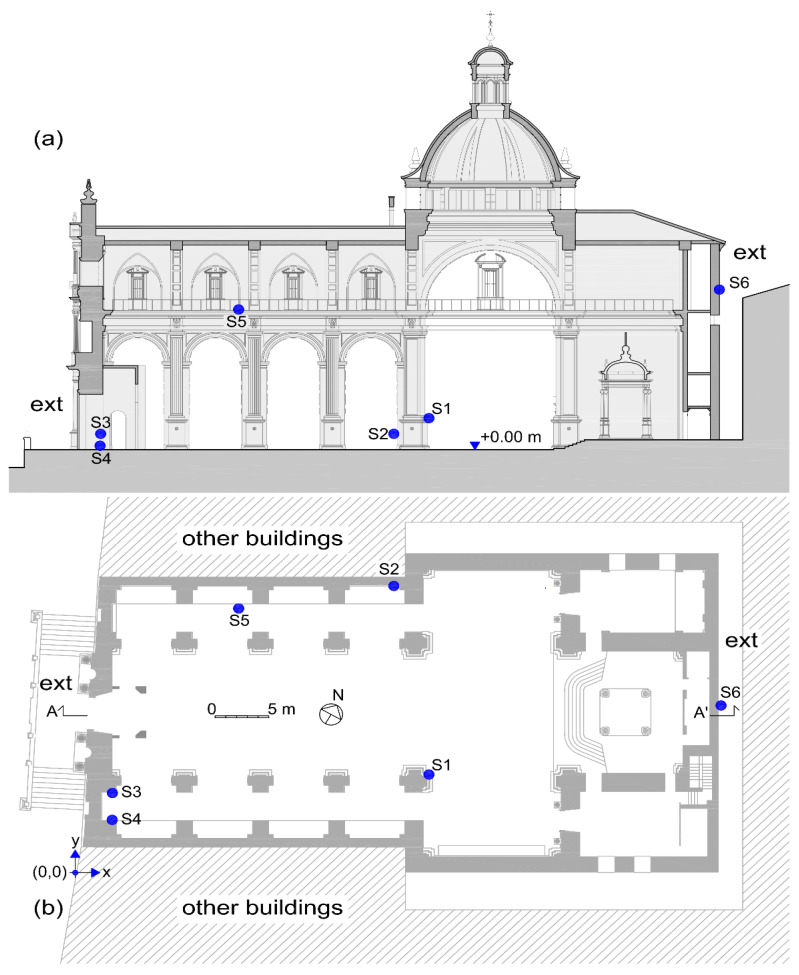
Sensors location in the building: (**a**) section A-A’ and (**b**) plan view.

The location of the sensors is indicated in Table 2 and Figure 2. The sensors have been sought to be in different orientations of the building, as well as at different heights.

### 2.3. Computational Fluid Dynamics (CFD) Simulation

CFD simulations are done with the software Ansys Fluent. The model comprises, with a simplified geometry, the entire volume of air inside the building, considering the openings of doors and windows as air inlets and outlets, wall arrangements, pillars, dome, etc.

In this research, the analysis has focused on the most extreme cases: summer and winter in order to make the contrasts more evident. The data used in the simulations as boundary conditions are shown in Table 3, taken from the sensors. The data for summer correspond to 9 August 2019 at 5:40 p.m. where the maximum outdoor temperature was reached and those for winter correspond to 20 January 2020 at 7:50 p.m. where the minimum outdoor temperature was reached.

**Figure 3:**
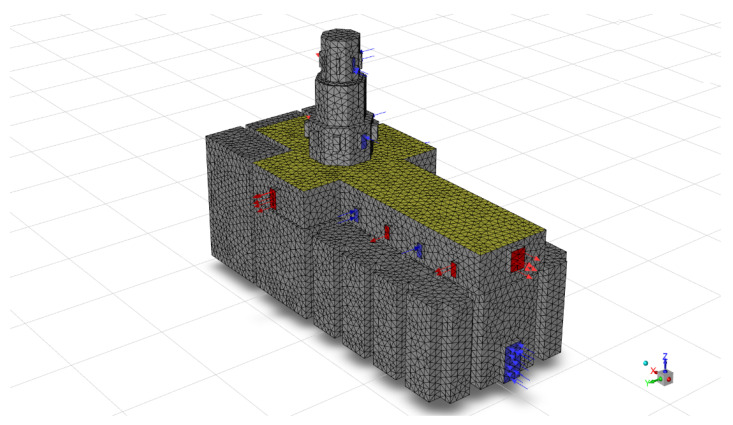
Finite element model used in calculations.

The finite element mesh has been created automatically by the software since the global study of the building did not consider local regions that require special analysis. Although a maximum size of the element is indicated to guarantee a sufficient number of nodes and that the precision when performing the calculations is optimal. In this sense, the model consists of approximately 36,108 total nodes and 189,590 elements. The minimum mesh is 1 × 1 m^2^ (Figure 3). The finite element model is a simplification of the real geometry of the building. Blue vectors indicate the velocity inlet flow and red vectors the velocity outlet one with CFD setup parameters.

**Figure 4:**
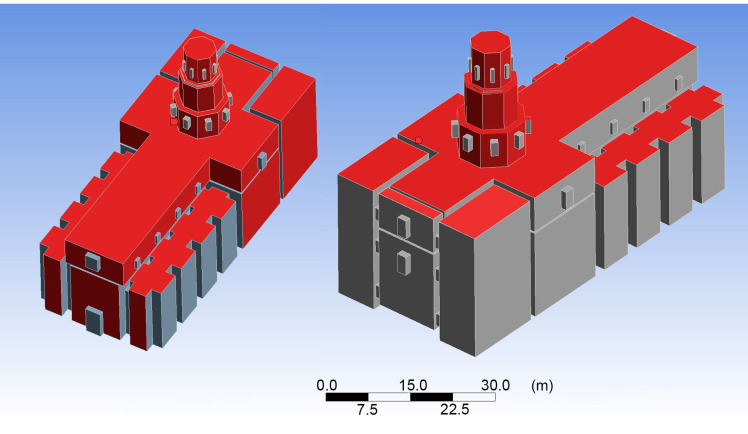
Surfaces with a high degree of exposure to the elements (red colour).

Due to the location of the building in the urban fabric, all the enclosures are not exposed to the outside and to sunlight with the same intensity. This has been taken into account in the calculations. In Figure 4, those faces of the model that are most exposed have been indicated in red.

The air volume of the interior of the building has been modelled, considering an average thickness of the facade walls of 85 cm with a calcareous stone material, since the walls are composed of masonry. In order to simulate both the temperature and the relative humidity of the air, a mixed material has been specified that takes into account the phase changes that occur with evaporation or condensation of the air as a function of temperature. Table 4 shows the values of the main properties of the materials used. Table 5 shows the setup details. The film coefficients used inside the building are 35 W·m^−2^·K^−1^ as it is a natural convection analysis, where the values are usually between 5–37 [49].

The physical models used in the calculations have been: Energy, Viscous (Realizable k-epsilon with standard wall function), Radiation (S2S) with solar load in Lliria (Valencia, Spain), and Species (Species Transport, to simulate relative humidity). In all the simulations calculated, no less than 500 iterations were executed, although the solution usually converges to less than 100.

Following the CFD software theory guide [26], conduction heat transfer is governed by Fourier’s Law (Equation (1)), where K is the thermal conductivity and ∇*T* is the temperature gradient. Fourier’s law states that the heat transfer rate is directly proportional to the gradient of temperature. The software computes conduction in all fluid and solid zones when the energy equation is activated.

(1) $$q_{conduction}=-K\nabla T$$

Convection, which simulates the natural convection of the air (Equation (2)) and radiation, which simulates the radiation of the environment (Equation (3)), is considered for the thermal condition of the walls. Here, *h_ext_* is the outside heat transfer coefficient, *T_ext_* the outside temperature, *T_w_* wall temperature, external emissivity *ε_ext_*, and *σ* the Stefan-Boltzmann constant (5.6704 × 10^−8^ W·m^−2^·K^−4^).

(2) $$q_{conv}=h_{ext}\left(T_{ext}-T_{w}\right)$$

(3) $$q_{rad}=\varepsilon_{ext}\sigma \left(T_{\infty }^{4}-T_{w}^{4}\right)$$

For the combined study of these two parameters, a mixed system of boundary conditions is used (Equation (4)).

(4) $$q_{mixed}=h_{ext}\left(T_{ext}-T_{w}\right)+\varepsilon_{ext}\sigma \left(T_{\infty }^{4}-T_{w}^{4}\right)$$

Natural convection occurs when fluid density is temperature dependent and heat is added to the fluid. Flow is induced by gravitational force acting on density differences. When gravity is activated, the pressure gradient and body force terms in the momentum equation are rewritten as in Equation (5), with *p*’ = *p* − *p*_0_*gx*. The radiation model selected must be appropriate for the optical thickness of the system being simulated. Surface to surface model (S2S) is selected; when optical thickness is equal to zero, S2S has comparable accuracy with Discrete ordinates model (DO) at less computational expense.

(5) $$-\frac{\partial p}{\partial x}+pg\;\to \frac{\partial p^{\prime }}{\partial x}+\left(p-p_{0}\right)g$$

The energy equation is solved in the following form:

(6) $$\frac{\partial \left(\rho E\right)}{\partial t}+\nabla \cdot \left[V\left(\rho E+p\right)\right]=\nabla \cdot [K_{eff}\nabla T-\;\underset{j}{\sum }h_{j}J_{j}+\tau_{eff}\cdot V]+S_{h}$$

From Equation (6), $\frac{\partial \left(\rho E\right)}{\partial t}$ is the part of unsteady, ∇·[V(ρE+p)] is convection, $K_{eff}\nabla T$ is conduction, $\sum_{j}h_{j}J_{j}+\tau_{eff}\cdot V]+S_{h}$ is the species diffusion, $\tau_{eff}\cdot V$ is the viscous dissipation, and $S_{h}$ the enthalpy source.

In some other cases, the problem can be studied by separating it into two sections: the heat transfer in fluid, and the heat transfer in solid [50]. In this case, convection heat transfer results from fluid motion, where the heat transfer rate is coupled to the fluid flow solution; the rate of heat transfer is strongly dependent on fluid velocity and fluid properties, and fluid properties may vary significantly with temperature. Also, the velocity depends on the viscosity of the fluid [51].

### 2.4. Infrared Thermography (IRT)

In addition to the real data from the sensors, an IRT campaign is also set up inside the building. In this study, a FLIR E95 camera has been used. It produces thermographic pictures at a resolution of 464 × 348 pixels, with a temperature range of −20 to +120 °C and an accuracy of ±2 °C or ±2% of reading for ambient temperature 15 °C to 35 °C and object temperature above 0 °C [52].

The thermographic pictures were subsequently processed with FLIR Tools software, which can adjust the colour palette, temperature range, distance, as well as calculate the values of maximum, minimum, average temperatures in the selected areas and the specific temperature of the points where the sensors are located. All devices were appropriately calibrated before the measurements according to the operation manual. The emissivity values of the most common construction materials are over 90%, and in our study, have been taken 0.95, therefore, it is expected that the results obtained from thermographic measurements are reliable [53] and the emissivity is practically constant for non-metallic materials [54].

## 3. Results and Discussion

### 3.1. Real Data with Sensors

**Figure 5:**
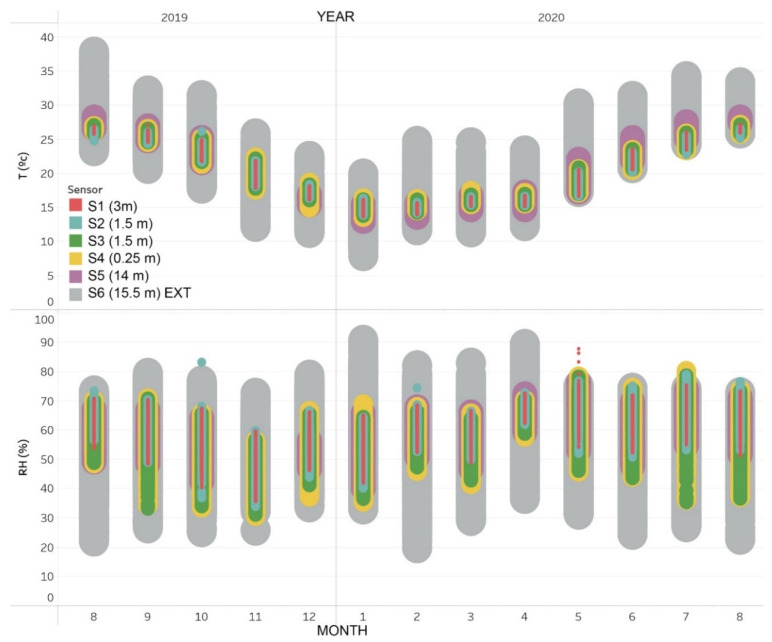
Overview of Temperature and Relative Humidity for each month.

The data recorded by the sensors have allowed a global vision of the temperature and relative humidity of the building for a year, between August 2019 and August 2020 (Figure 5). This figure shows that the outdoor temperatures are included in a wide range during all the months of the year, between 5 and 40 °C. However, for each month, the average temperature usually varies ±7 °C. The interior temperatures, however, are limited in a much smaller interval, ±5 °C with respect to the average of each month. This range of indoor temperatures is lower in the summer and winter months and is higher in the spring and fall months.

Regarding relative humidity, Figure 5 shows how the range of outdoor values is very wide in all months of the year. The range of relative humidity inside the building is also very wide and variable.

The average temperature T (°C) has been calculated for all the months of the year (Table 6). The minimum-maximum temperature gradient of the sensors inside the building has been graphed in a blue-red colour scale. It is observed that the months of June to October correspond to the hottest and the months of December to April to the coldest, adjusting to the Mediterranean climate.

Similarly, the average relative humidity RH (%) for each month is shown in Table 7. The relative humidity data outside the building (sensor S6) indicates that in the hot period the values were lower (56.2% in June) and higher in the winter months (63.1% in January). However, inside the building, the months of April to September have registered values of relative humidity clearly higher than the months of October to January. It should be borne in mind that due to the Sars-Cov-2 virus and the disease that causes COVID-19, in Spain there was confinement, mobility restrictions, and limitation of religious acts with a significant number of attendees (such as masses, in this case) since the end of March 2020, so the building was closed and unused until July. This coincides with the increase in relative humidity that can be seen during these months.

Likewise, the accumulated precipitation each month is shown with data from AEMET (State Meteorological Agency of Spain) [55] in order to observe the characteristic rain periods (Table 8). The months from January to April, except February, have been the rainiest.

Various authors [7,19] have tried to establish criteria to identify the areas of buildings or the most significant moments in which condensation may occur and increase the risk of deterioration of construction materials. There are three parameters that indicate a greater risk of condensation: (i) temperature, since when it is low there is a greater probability that condensation will occur; (ii) the difference between the temperature T and the dew temperature T_D_, since condensations occur if T_D_ > T; (iii) and the renewal of air in the space to be studied, since an air renewal reduces the saturation of water vapor and, therefore, reduces condensation.

With these premises, a Risk index (Equation (7)) has been established to combine the three parameters. Factor (ii) is considered the most determining and has been analysed with *α* = 50% of the score. Factor (i) is also important and is valued with *β* = 40%. Factor (iii) is valued with *γ* = 10% due to the little influence of air renewal in a building with such a large volume of air.

(7) $$Risk\;Index=\alpha \cdot R_{T}+\beta \cdot R_{D}+\gamma \cdot R_{V}$$

where *R_T_* is the risk of condensation due to ambient temperature, assigning in this case the value 1 at 0 °C and the value 0 at 40 °C and interpolating linearly for intermediate values. *R_D_* is the risk of condensation due to the proximity of the ambient temperature with the dew temperature, assigning the value 1 for T − T_D_ = 0 and a value of 0 for T − T_D_ = 30 and interpolating linearly. Finally, *R_V_* is the risk of condensation due to air renewal, assigned a value of 0.9 during confinement months (April to July), 0.75 to very cold or very hot months (August, September, December, January, February, March) where ventilation is not provided due to weather conditions, and 0.5 points to the rest of the months (October and November).

**Figure 6:**
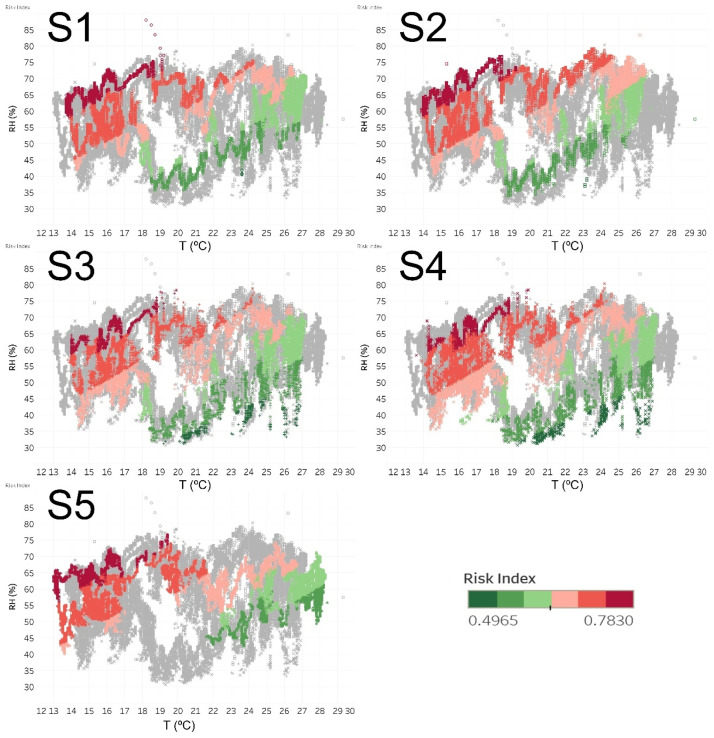
Comparison of the Risk index for each sensor (colour scale) on the Temperature–Relative Humidity graph.

Table 9 shows how the risk of condensation inside the building is higher in the months of January to May, but especially in April, coinciding with relatively low temperatures, high relative humidity and little air renewal due to the restrictions of mobility imposed by COVID-19. Figure 6 shows in colour scale (green-red) the risk of condensation for each of the five sensors inside the building. It is a graph that relates temperature (T (°C)) and relative humidity (RH (%)). In addition, the data of each sensor is superimposed on the data of all the sensors (grey colour) to be able to compare each sensor and to know which areas of the graph it covers.

### 3.2. Computational Fluid Dynamics (CFD) Simulation

The most extreme cases have been simulated, such as the hottest day (maximum outside temperature) and the coldest day (minimum outside temperature).

**Figure 7:**
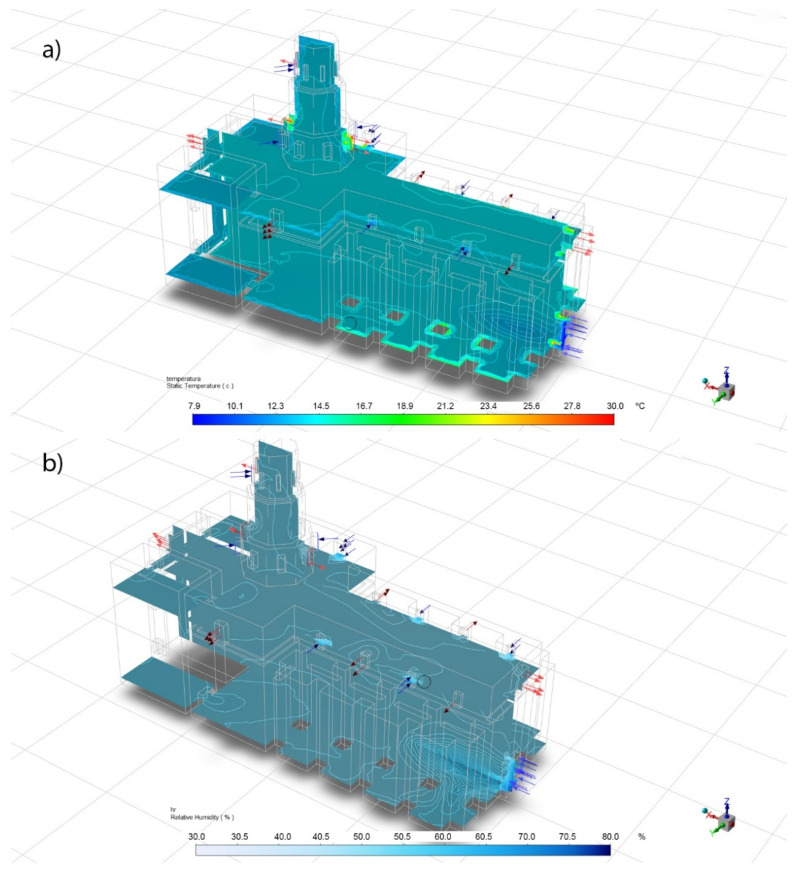
Winter CFD simulation perspective. (**a**) Temperatures and (**b**) relative humidity.

The winter case occurred on 20 January 2020 at 8:00 p.m., with an outdoor temperature of 7.9 °C and a RH 78.8%, data that have been used as boundary conditions (BC). With these data, in Figure 7 the temperature and relative humidity inside the building have been simulated. Three planes are graphed: longitudinal ZX plane along the axis of the building and two horizontal XY planes (1 m and 16 m high). The values are maintained with little variation, except in the vicinity of the openings of doors and windows, as well as the walls.

Table 10 shows the comparison between the temperature and relative humidity data recorded by the sensors and those obtained in the CFD simulation for the winter case. Thus, the table shows, for each sensor, the value of the temperature and relative humidity recorded and the value obtained in the CFD simulation at the same location. In this case, the software provides a confidence interval and not an exact value. Also, the difference is showed if the sensor value is not in the confidence interval of the CFD. For temperatures, the biggest difference is 0.03 °C. In the case of relative humidity, the biggest difference is 1.3%. In both cases, the simulation data is very accurate.

**Figure 8:**
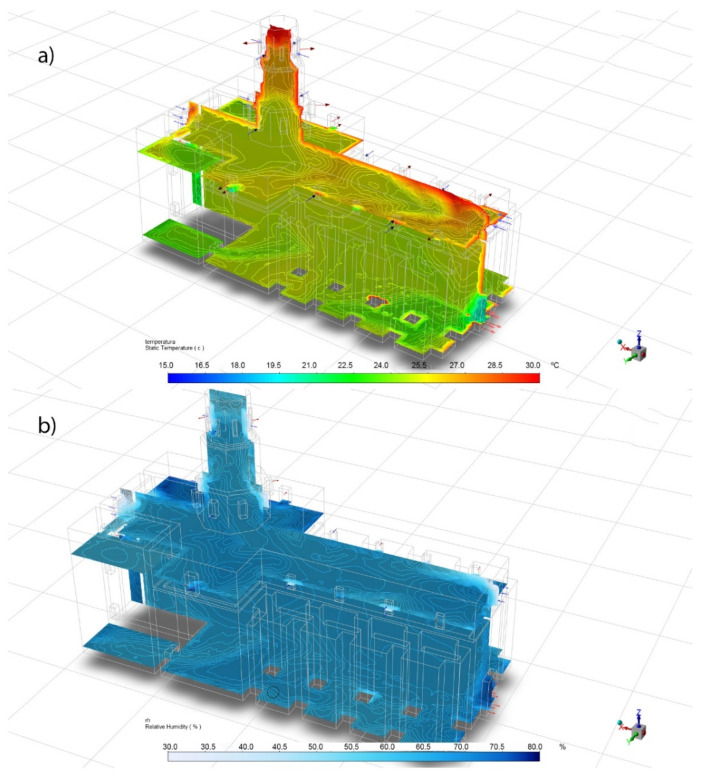
Summer CFD simulation perspective. (**a**) Temperatures and (**b**) relative humidity.

The case studied for summer happened on 9 August 2019 at 5:40 p.m., with an outdoor temperature of 37.9 °C and a RH of 22.6%, data that have been used as boundary conditions. With these data, in Figure 8 the temperature and relative humidity inside the building have been simulated. On this occasion, the sunlight and the different surfaces of the building that are more or less exposed to the elements cause more significant temperature differences than in the case of winter. The upper area of the roof and the south and west facades have higher temperatures, unlike other areas in the east and north, which are more protected.

Table 11 shows the comparison between the temperature and relative humidity data recorded by the sensors and those obtained in the CFD simulation for the summer case. Also, the difference is showed if the sensor value is not in the confidence interval of the CFD. For temperatures, the biggest difference is 1.15 °C for the S1 sensor, although in the rest the differences are much smaller. In the case of relative humidity, the greatest difference is 7.9% for the S2 sensor, but for the rest the differences are minimal (less than 1%). Therefore, in both cases, the simulation data is also very accurate.

These real values that differ from what is expected from CFD simulations should be studied carefully because they could indicate critical zones or moments.

**Figure 9:**
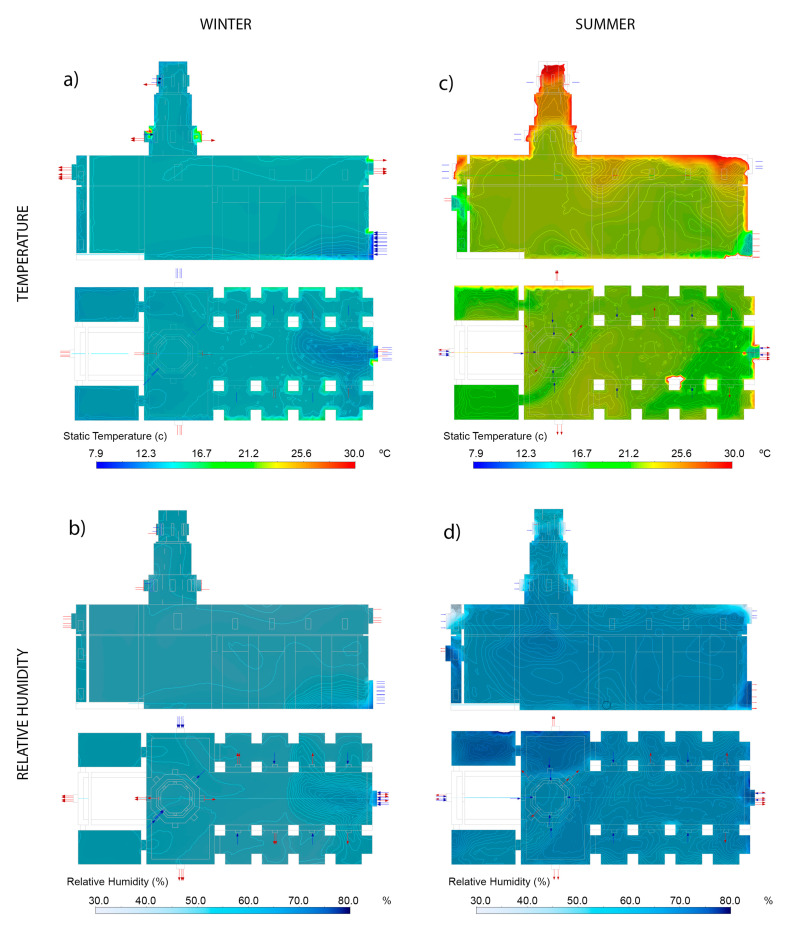
Comparison of CFD simulations. (**a**) T (°C) in winter, (**b**) RH (%) in winter, (**c**) T (°C) in summer, (**d**) RH (%) in summer.

Figure 9 shows a comparison of the CDF simulations for the summer and winter cases studied, both for temperature and relative humidity. In addition, the horizontal XY plane is shown at a height of 1 m and the longitudinal ZX plane along the axis of the building. This comparison allows to analyse, qualitatively, what is the hygrothermal situation in the building in each case.

### 3.3. Infrared Thermography (IRT)

**Figure 10:**
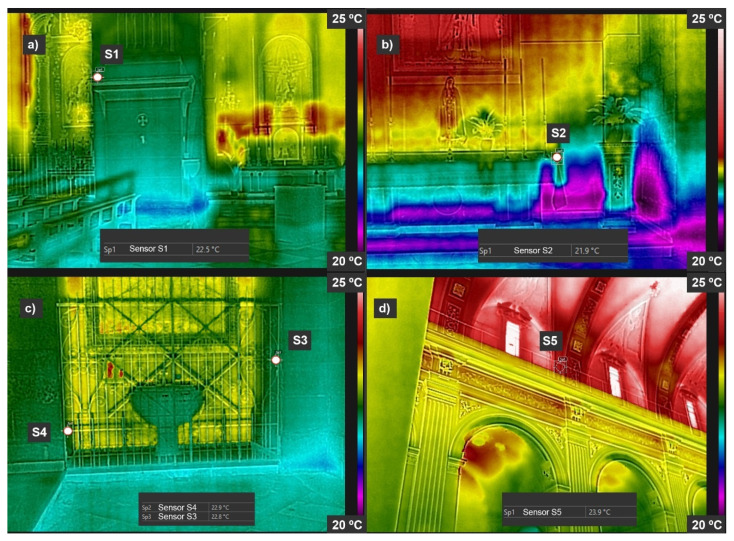
IRT pictures. (**a**) Sensor S1, (**b**) sensor S2, (**c**) sensors S3 and S4, (**d**) sensor S5.

The thermograms shown in Figure 10 were taken on 24 June 2020 between 10:00 and 11:00 a.m. The points marked in the figure indicate the position of the sensors. Thermographic pictures show the temperature distribution of the different surfaces. Likewise, the figure shows the temperature registered by the IRT in the region where each of the sensors is located inside the building.

**Figure 11:**
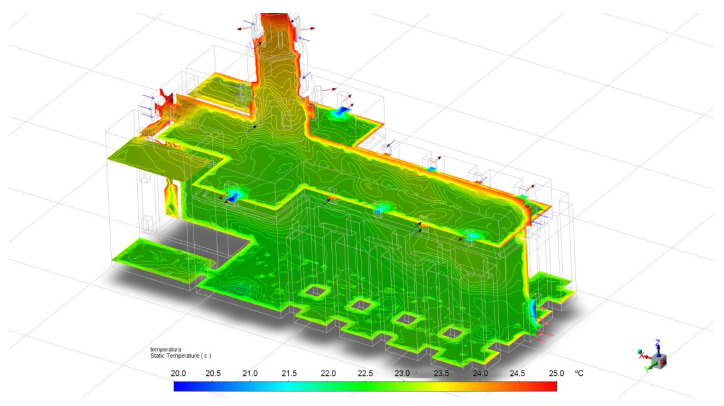
CFD simulation to compare results with sensors and IRT pictures.

In Figure 11, the CFD simulation is shown. In this case, the same temperature and relative humidity conditions were used as when the IRT pictures were taken, with the real values recorded by the sensors. These real values from the thermograms allow to complete the validation of the sensors and the CFD simulations. Table 12 compares the temperature values obtained by the sensors and those recorded by the CFD simulation and the IRT pictures, as well as the precision achieved in each case. Also, the difference is showed if the sensor value is not in the confidence interval of the CFD. It is observed that the differences between the temperature values of the sensors and those obtained by the CDF simulation are not greater than 0.5 °C. In the case of the temperatures recorded by infrared thermography, the differences with respect to the values obtained by the sensors are also very small, not greater than 0.3 °C.

## 4. Conclusions

This work has focused on the hygrothermal analysis of a heritage building over a period of 1 year using three NDTs: temperature and relative humidity data-loggers sensors, IRT thermographic pictures and CFD finite element simulations.

The proposed methodology consists of recording temperature and relative humidity values with sensors distributed at strategic points in the building. These actual values allow to verify and validate the CFD simulations, which cover the entire interior volume of the building. Likewise, infrared thermography allows to complete the verification of the simulations.

The more than 200,000 values of temperature and relative humidity registered by the sensors allow to obtain a clear vision of the hygrothermal evolution of the building. Outdoor temperatures are in a wide range during all months of the year, between 5 and 40 °C. However, the interior temperatures are limited to a smaller interval, ±7 °C with respect to the average temperature of each month. Also, this range of indoor temperatures is lower in the summer and winter months and is higher in the spring and autumn months.

The proposed methodology seeks to identify those areas of the building and when there is a high risk of condensation, which can affect the integrity of building materials and the healthiness of the interior environment. A Risk Index is established based on the temperature, the difference between temperature with respect to the dew temperature and the renewal of the air inside the building. For the case studied, the risk of condensation inside the building is greater in the months of January to May, but especially in April, coinciding with relatively low temperatures, high relative humidity and little air renewal due to the mobility restrictions imposed by COVID-19. However, in addition, when the height is greater inside the building, the risk of condensation increases.

The CFD simulations include the most representative physical models to represent the case studied, which includes energy, radiation with solar load, and phase changes in air to simulate relative humidity. The most representative cases, summer and winter, have been studied. The sections of the building generated in the simulations allow a first qualitative analysis of temperatures and relative humidity. A quantitative analysis has also been carried out by comparing the temperature and relative humidity data recorded by the sensors and those obtained in the CFD simulation. In the case of winter, the difference between the temperatures is 0.03 of a °C. In the case of relative humidity, the biggest difference is 1.3%. For the summer case, the largest temperature difference between the sensors and the CFD is 1.15 °C for the S1 sensor, although in the rest the differences are much smaller. In the case of relative humidity, the biggest difference is 7.9% for the S2 sensor, but for the rest, the differences are also minimal (less than 1%). Therefore, in both cases, the simulation data is very accurate.

Infrared thermography has completed the verification of CFD simulations. For this, the conditions when the thermographic images were taken have been modelled and the temperature values of the sensors, CFD and IRT have been compared. The differences between the temperature values of the sensors and those obtained by the CDF simulation are not greater than 0.5 °C. In the case of the temperatures recorded by infrared thermography, the differences with respect to the values obtained by the sensors are also very small, not greater than 0.3 °C.

Future works may address different applications of this methodology in heritage buildings, which are highly susceptible to humidity and condensation. A greater number of sensors will allow a better sensitivity of the hygrothermal analysis and greater control points of the CFD simulations. Even a transient study using CFD over time can be interesting to be able to be compared with the average data from the sensors. Likewise, an IRT campaign on different days of the year will provide more information and values to confront, achieving a better knowledge of reality and a better conservation of our heritage.

## Figures and Tables

**Table 1:** Main characteristics of the data-loggers.

Feature	Value
Temp. measurement range	40 °C to 125 °C
Humidity measurement range	0 to 100% RH
Max. capacity	21,000 values
Temp. measurement accuracy	±0.3 °C @25 °C
Humidity measurement accuracy	±2% RH @25 °C

**Table 2:** Location of the data-loggers in the building.

Sensor	Location	Orientation	Coordinates (x, y)	Height (m)
S1	Int	-–-	31.25, 7.13	+3.00
S2	Int	North	28.82, 25.33	+1.50
S3	Int	West	2.41, 6.28	+1.50
S4	Int	South-West	2.41, 3.22	+0.25
S5	Int	North	13.55, 23.98	+14.00
S6	Ext	East	56.85, 13.21	+15.50

**Table 3:** Real data from the sensors used for the simulations.

Sensor	Winter		Summer	
	Temperature (°C)	RH (%)	Temperature (°C)	RH (%)
S1 (3 m)	14.1	53.2	26.4	64.5
S2 (1.5 m)	14.3	52.3	25.3	68.5
S3 (1.5 m)	14.1	51.4	26.4	62.1
S4 (0.25 m)	14.1	52.1	26.4	63.9
S5 (14 m)	13.4	52.1	27.9	50.9
S6 (15.5 m) EXT	7.9	78.8	37.9	22.6

**Table 4:** Physical properties of the materials used.

Property	Mixture Air	Stone
Density (kg·m^−3^)	incompressible	2800
Viscosity (kg·m^−1^·s^−1^)	1.72 × 10^−5^	-
Conductivity (W·m^−1^·K^−1^)	0.0454	2.25
Specific heat (J·kg^−1^·K^−1^)	1006.43	856
Emissivity	1	0.92

**Table 5:** Setup details of CFD simulations.

Location	Type	Details
Ceiling, Floor, Walls	Wall	No-slip shear condition, stationary wall, with roughness constant of 0.5.
Air	Fluid	Mixture material with species (H_2_O, air).
Inlet holes	Inlet	Velocity magnitude: 0.05 m·s^−1^. Turbulent intensity: 5%, Turbulent viscosity ratio: 10.
Outlet holes	Outlet	Gauge pressure (pascal): 0. Backflow turbulent intensity: 5%. Backflow turbulent viscosity ratio: 10

**Table 6:** Average temperature T (°C) registered by the sensors.

Sensor (Heigh)	Jan	Feb	Mar	Apr	May	Jun	Jul	Aug	Sep	Oct	Nov	Dec
S1 (3 m)	14.5	15.0	16.0	15.8	18.9	21.9	24.9	26.6	25.4	23.6	19.7	17.0
S2 (1.5 m)	14.8	15.1	16.0	15.9	18.6	21.5	24.2	25.7	25.0	23.3	19.8	17.1
S3 (1.5 m)	14.7	15.4	16.3	16.1	19.0	21.9	24.9	26.6	25.5	23.7	19.8	17.1
S4 (0.25 m)	15.0	15.5	16.5	16.4	19.1	21.9	24.8	26.6	25.6	23.8	20.0	17.2
S5 (14 m)	13.8	14.9	15.9	15.8	20.1	23.3	26.4	27.7	25.7	24.1	19.5	16.2
S6 (15.5 m) EXT	11.9	15.1	15.2	16.2	22.1	24.7	27.9	28.6	25.1	22.3	16.9	14.8

**Table 7:** Average relative humidity RH (%) registered by the sensors.

Sensor (Heigh)	Jan	Feb	Mar	Apr	May	Jun	Jul	Aug	Sep	Oct	Nov	Dec
S1 (3 m)	55.2	62.9	59.8	67.9	69.1	65.6	69.9	65.0	62.7	55.7	45.9	55.3
S2 (1.5 m)	54.3	62.7	59.8	67.9	70.5	67.3	72.5	68.0	63.9	56.4	45.4	54.5
S3 (1.5 m)	52.1	59.7	56.4	65.2	66.7	63.2	67.9	62.1	59.4	52.0	42.1	52.3
S4 (0.25 m)	51.8	59.4	56.4	65.0	67.3	64.0	68.8	62.2	59.3	51.9	41.8	51.8
S5 (14 m)	54.8	61.4	58.3	66.3	64.8	60.7	65.0	60.4	59.9	53.6	46.6	52.6
S6 (15.5 m) EXT	63.1	60.8	60.7	68.7	57.8	56.2	58.7	56.5	61.0	55.3	48.9	59.4

**Table 8:** Rain (mm). Source: AEMET (State Meteorological Agency of Spain).

	Jan	Feb	Mar	Apr	May	Jun	Jul	Aug	Sep	Oct	Nov	Dec
Precipitation	105.4	2.0	94.8	81.4	23.6	0.0	8.8	59.4	56.8	20.4	4.6	38.8

**Table 9:** Risk index of condensation inside the building.

Sensor (Heigh)	Jan	Feb	Mar	Apr	May	Jun	Jul	Aug	Sep	Oct	Nov	Dec
S1 (3 m)	0.72	0.74	0.72	0.76	0.73	0.69	0.67	0.62	0.63	0.60	0.60	0.69
S2 (1.5 m)	0.71	0.74	0.72	0.76	0.74	0.70	0.68	0.64	0.63	0.60	0.60	0.69
S3 (1.5 m)	0.71	0.72	0.70	0.75	0.72	0.68	0.66	0.61	0.61	0.58	0.58	0.68
S4 (0.25 m)	0.70	0.72	0.70	0.74	0.72	0.68	0.66	0.61	0.61	0.58	0.58	0.68
S5 (14 m)	0.72	0.73	0.71	0.75	0.70	0.66	0.64	0.59	0.61	0.58	0.59	0.69

**Table 10:** Comparison of temperature and relative humidity in the case of winter.

Sensor (Heigh)	T (°C) Sensor	T (°C) CFD	Accuracy (°C)	RH (%) Sensor	RH (%) CFD	Accuracy (%)
S1 (3 m)	14.1	14.08–14.30	In	53.2	51–52	−1.2
S2 (1.5 m)	14.3	14.31–14.53	+0.01	52.3	50–51	−1.3
S3 (1.5 m)	14.1	14.08–14.30	In	51.4	50–51	−0.4
S4 (0.25 m)	14.1	13.86–14.08	−0.03	52.1	51–52	−0.1
S5 (14 m)	13.4	13.43–13.65	+0.03	52.1	53–54	+0.9
S6 (15.5 m) EXT	7.9	7.9	In	78.8	78.8	In

**Table 11:** Comparison of temperature and relative humidity in the case of summer.

Sensor (Heigh)	T (°C) Sensor	T (°C) CFD	Accuracy T (°C)	RH (%) Sensor	RH (%) CFD	Accuracy RH (%)
S1 (3 m)	26.4	25.00–25.25	−1.15	64.5	63.30–63.75	−0.75
S2 (1.5 m)	25.3	25.00–25.25	−0.05	68.5	60.15–60.60	−7.90
S3 (1.5 m)	26.4	26.50–26.75	+0.10	62.1	61.50–61.95	−0.15
S4 (0.25 m)	26.2	26.25–26.50	+0.05	60.9	60.15–60.60	−0.30
S5 (14 m)	27.9	28.25–28.50	+0.35	50.9	50.25–50.75	−0.15
S6 (15.5 m) EXT	37.9	37.9		22.6	22.6	

**Table 12:** Comparison of temperature recorded by sensors, CFD and IRT.

Sensor (Heigh)	T (°C) Sensor	T (°C) CFD	Accuracy CFD T (°C)	T (°C) IRT	Accuracy IRT T (°C)
S1 (3 m)	22.7	22.70–22.75	In	22.5	−0.2
S2 (1.5 m)	22.2	22.45–22.50	+0.25	21.9	−0.3
S3 (1.5 m)	22.7	22.65–22.70	In	22.8	+0.1
S4 (0.25 m)	22.7	22.70–22.75	In	22.9	+0.2
S5 (14 m)	24.2	23.70–23.75	−0.50	23.9	−0.3
S6 (15.5 m) EXT	24.9	24.9			
